# Association mapping of a locus that confers southern stem canker resistance in soybean and SNP marker development

**DOI:** 10.1186/s12864-019-6139-6

**Published:** 2019-10-31

**Authors:** João Vitor Maldonado dos Santos, Everton Geraldo Capote Ferreira, André Luiz de Lima Passianotto, Bruna Bley Brumer, Adriana Brombini Dos Santos, Rafael Moreira Soares, Davoud Torkamaneh, Carlos Alberto Arrabal Arias, François Belzile, Ricardo Vilela Abdelnoor, Francismar Corrêa Marcelino-Guimarães

**Affiliations:** 1 Brazilian Agricultural Research Corporation, National Soybean Research Center (Embrapa Soja), Carlos João Strass Road, Warta County, PR Brazil; 2 Londrina State University (UEL), Celso Garcia Cid Road, km 380, Londrina, PR Brazil; 3 Present address: Department of Plant Agriculture, University of Guelph, Guelph, Ontario N1G 2V7 Canada; 4 Department of Plant Sciences and Institute of Integrative Biology and Systems (IBIS), Université Laval, Quebec City, G1V 0A6 Canada

**Keywords:** *Diaporthe aspalathi*, GWAS, Haplotype analysis, Marker assisted selection (MAS)

## Abstract

**Background:**

Southern stem canker (SSC), caused by *Diaporthe aspalathi* (E. Jansen, Castl. & Crous), is an important soybean disease that has been responsible for severe losses in the past. The main strategy for controlling this fungus involves the introgression of resistance genes. Thus far, five main loci have been associated with resistance to SSC. However, there is a lack of information about useful allelic variation at these loci. In this work, a genome-wide association study (GWAS) was performed to identify allelic variation associated with resistance against *Diaporthe aspalathi* and to provide molecular markers that will be useful in breeding programs.

**Results:**

We characterized the response to SSC infection in a panel of 295 accessions from different regions of the world, including important Brazilian elite cultivars. Using a GBS approach, the panel was genotyped, and we identified marker loci associated with *Diaporthe aspalathi* resistance through GWAS. We identified 19 SNPs associated with southern stem canker resistance, all on chromosome 14. The peak SNP showed an extremely high degree of association (*p*-value = 6.35E-27) and explained a large amount of the observed phenotypic variance (*R*^*2*^ = 70%). This strongly suggests that a single major gene is responsible for resistance to *D. aspalathi* in most of the lines constituting this panel. In resequenced soybean materials, we identified other SNPs in the region identified through GWAS in the same LD block that clearly differentiate resistant and susceptible accessions. The peak SNP was selected and used to develop a cost-effective molecular marker assay, which was validated in a subset of the initial panel. In an accuracy test, this SNP assay demonstrated 98% selection efficiency.

**Conclusions:**

Our results suggest relevance of this locus to SSC resistance in soybean cultivars and accessions from different countries, and the SNP marker assay developed in this study can be directly applied in MAS studies in breeding programs to select materials that are resistant against this pathogen and support its introgression.

## Background

Cultivated soybean [*Glycine max* (L.) Merrill] is one of the most important crops worldwide. It has been estimated that wild soybean (*Glycine soja*) was domesticated to cultivated soybean approximately 7000–9000 years ago in Asia but reached the Americas only on the eighteenth century [1]. Currently, the Americas are responsible for 90% of the world’s soybean production. In Brazil, soybean is a major agricultural commodity, showing production of 119 M tons from 35 M hectares of cultivated land in the 2017/18 growing season [2]. Due to its major importance to the Brazilian economy, a large number of studies have been undertaken to better understand genetic variation in the soybean genome and its relationship to traits of interest [3].

An important barrier to increased soybean production and seed quality is the large number of biotic factors that affect soybean production. One of the main pathogens responsible for considerable losses in soybean fields is southern stem canker (SSC). SSC is caused by the fungus *Diaporthe aspalathi*, anamorph *Phomopsis aspalathi* (Cooke & Ellis), belonging to the *Diaporthe/Phomopsis* complex, which is associated with other diseases in soybean such as seed decay and pod and stem blight. Historically, two causal agents of SSC have been described: *Diaporthe phaseolorum* var*. meridionalis* (*Dpm*) F.A. Fernández and *Diaporthe phaseolorum* var. *caulivora* (*Dpc*) K. L. Athow & R. M. Caldwell. Recently, the names of these species (*Dpm* and *Dpc*) have been changed to *Diaporthe aspalathi* (E. Jansen, Castl. & Crous) (*Da*) and *Diaporthe caulivora* (Athow & Caldwell) J.M. Santos, Vrandecic & A.J.L. Phillips (*Dc*), respectively [4–6].

The *Da* fungus was reported for the first time in Brazil during the 1989/90 soybean cropping season in the states of Paraná and Mato Grosso, and in the following cropping season, SSC was observed in almost all soybean production areas in the country [7, 8]. In 1994, SSC was responsible for losses of 1.8 million metric tons in Brazil, making it the most serious disease of the Brazilian soybean crop at that time [9].

Currently, genetic resistance is the main method of SSC control, and most of the cultivars being cropped carry SSC resistance genes. To date, five major dominant, non-allelic SSC resistance loci (*Rdc1*, *Rdc2*, *Rdc3*, *Rdc4* and *Rdc5*) have been reported [10, 11]. Another source of resistance, distinct from *Rdc1–4*, was identified in PI 398469 and has provisionally been named *Rdc?* [12]. However, these loci were identified using *Da* isolates from the southern United States*,* and according to other studies, genes that confer resistance to one pathogen do not confer resistance to another [12, 13]. Therefore, it was proposed to rename the major loci related to *Da* resistance *Rdm1*, *Rdm2*, *Rdm3*, *Rdm4*, and *Rdm5* [13, 14]. Recently, *Rdm4* and *Rdm5* were mapped close together on chromosome 08 in the cultivar (cv.) Hutcheson [15]. Knowledge associated with the accurate localization of major genes responsible for host plant resistance to a pathogen is an important step in the identification of molecular markers that may be helpful in the development of cultivars resistant to SSC. In this context, genome-wide association studies (GWAS) offer great opportunity for identifying these resistance genes as well markers associated with resistance, representing an important tool for breeding programs.

The advent of new platforms for large-scale sequencing associated with the complete sequencing of the soybean genome [16] has allowed the genome-wide identification of a great number of variations that can be used to both characterize nucleotide and structural diversity in collections of soybean accessions and perform GWAS. A large number of GWAS are already available for soybean. Hwang et al. [17] identified 40 single nucleotide polymorphisms (SNPs) associated with protein content in 17 different genomic regions. In their study, 25 SNPs in 13 genomic regions were related to the control of oil content. Two different studies identified QTLs associated with resistance to *Sclerotinia sclerotiorum* [18, 19]. Mamidi et al. [20, 21] performed two studies on iron deficiency chlorosis (IDC). Contreras-Soto [22] identified 17, 59 and 11 SNPs associated with 100-seed weight, plant height and seed yield, respectively, using a panel of 169 soybean cultivars.

Despite the emergence of a large number of GWAS, many of these studies have been carried out using SNPs obtained via a genotyping by sequencing (GBS) approach and may therefore not have ensured full coverage of the soybean genome. Improved marker coverage can be achieved using whole-genome sequencing (WGS) data, and such exhaustive data can be useful for identifying and refining regions identified by GWAS performed with SNPs from GBS. For example, Zhou et al. [23] identified associations in 10 selected regions and 13 previously uncharacterized agronomic loci for characters including pubescence form, plant height, and oil content. Maldonado dos Santos et al. identified 5.8 million SNPs and 1.3 million InDels in 28 Brazilian soybean cvs. That could be used as a complementary source of information in GWAS. Valliyodan et al. [24] detected over 10 million SNPs in 106 soybean genomes, some of which were associated with oil and protein content, salinity, and domestication traits. Recently, a genome-wide study was developed in which two genes showing relevant associations with a soybean seed permeability trait were identified in *Glycine max* and *Glycine soja* [25]. These studies highlighted great power of whole-genome sequencing technologies for GWAS.

SSC is mainly controlled by the introgression of resistance genes in elite cultivars, and these genes are present in most cultivars released over the last 20 years in Brazil. However, the potential for considerable damage remains if current resistance genes are overcome by the pathogen. Thus, the molecular characterization of SSC resistance loci in a diverse set of soybean germplasms is essential to understand the genetic basis of SSC resistance. Therefore, the objective of this study was to identify allelic variation associated with resistance against *Da* in a diverse panel including soybean cultivars with a broad distribution and plants resulting from introductions in different regions of the world.

## Results

### Phenotypic evaluation of southern stem canker resistance in soybean accessions

All accessions were inoculated with mycelium from the CMES 480 isolate using the toothpick method under greenhouse conditions [26, 27]. The results of the inoculation experiment were expressed as the percentage of dead plants (%DPs), and all the differential genotypes showed a small lesion at the point on the stem where the toothpick penetrated, indicating that an infection had successfully occurred in all the inoculated plants. The cultivars Tracy-M (*Rdm1*/*Rdm2*), Crockett (*Rdm3*) and Hutcheson (*Rdm5*), which are sources of SSC resistance, showed complete resistance against the *D. aspalathi* isolate CMES 480, PI 398469 (*Rdm*?) also showed a high degree of resistance, but we still observed 3% DPs. On the other hand, the interactions between CMES 480 and the accessions harboring the *Rdm1* (D85–10404), *Rdm2* (D85–10412) and *Rdm4* (cv. Dowling) genes were all compatible, such that these accessions were all highly susceptible (Table 1). The isolate CMES 480 was recognized by multiple R genes, resulting in the possibility of identifying different resistance loci if they are distributed in the GWAS panel.

**Table 1:** Differential response of soybean genotypes to the CMES-480 southern stem canker isolate

Accession	Resistance Gene	%DP	SSC Phenotype
Tracy-M	*Rdm1*/*Rdm2*	0%	Resistant
D8510404	*Rdm1*	65%	Susceptible
D8510412	*Rdm2*	72%	Susceptible
Crockett	*Rdm3*	0%	Resistant
Dowling	*Rdm4*	56%	Susceptible
Hutcheson	*Rdm5*	0%	Resistant
PI398469	*Rdm?*	2%	Resistant

%DPs: The percentages of dead plants were obtained according to the formula proposed by Yorinori (1991) [27]

**Fig. 1:**
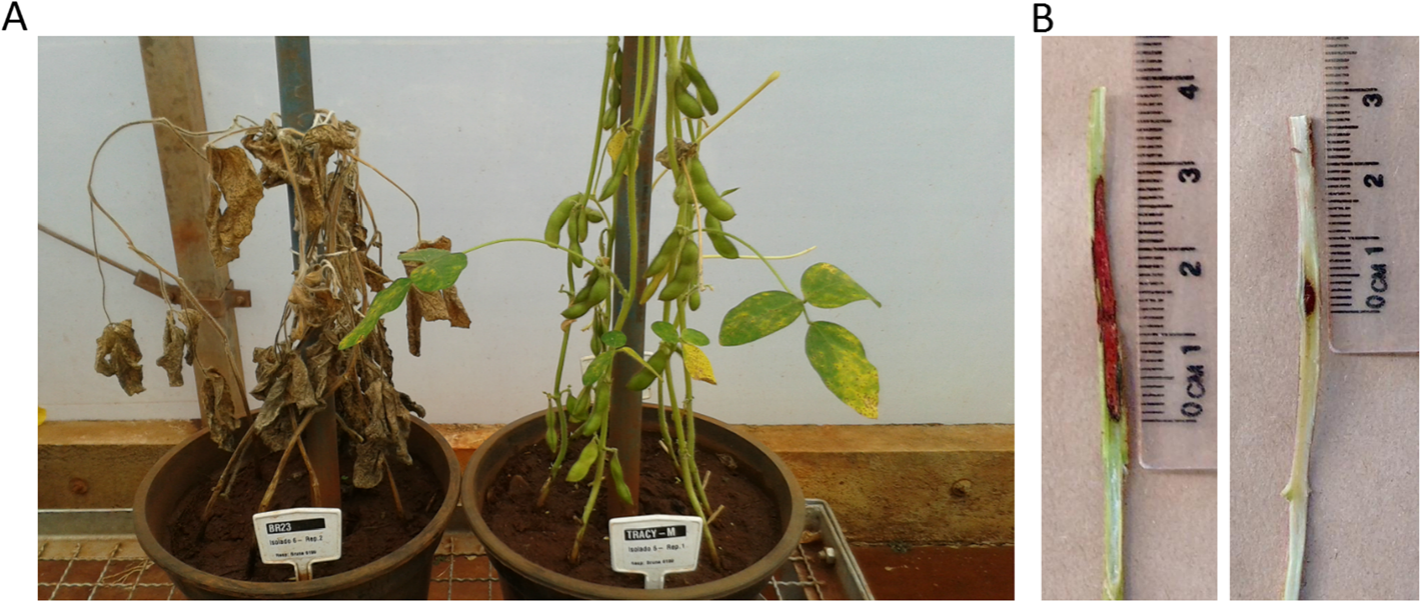
Phenotypic response to southern stem canker infection in soybean. **a** Differences between resistant (Tracy-M) and susceptible (BR-23) cultivars. **b** Lesion length in susceptible (left) and resistant (right) soybean accessions

Southern stem canker symptoms were evaluated at 60 days after inoculation and, as expected, known resistant (cv. Tracy-M) and susceptible (cv. BR 23) accessions showed highly contrasting results (Fig. 1a). The resistant plants showed only a small area of necrosis in the stem tissue around the toothpick, the presence of a callus at the toothpick insertion point and no damage to plant development. On the other hand, the susceptible accessions presented both infected and dead plants, where the infected plants were identified on the basis of the absence of a callus, a reduction in the development of the aerial parts of the plant, a large necrotic region at the point of inoculation, and the presence of chlorotic and withered plants. Another parameter that easily distinguished resistant and susceptible plants was the length of the internal lesion; resistant plants usually showed a lesion length of less than 1 cm, unlike susceptible plants, which presented lesions greater than 1 cm (Fig. 1b**).**

The pathogenicity test was carried out for all 295 accessions included in the GBS panel, where 205 were considered resistant, and 90 were susceptible. To highlight the diversity of the panel, among the resistant plants, 26% of the accessions came from China, 22% from Brazil, 20% from Japan and 12% from the USA. In the susceptible group, Brazil contributed 33% of the susceptible accessions; the USA contributed 20%; China contributed 18%; and South Korea contributed 17%. Based on the year of the release/cataloguing of the materials, the oldest resistant accessions in the panel (1930s) came from China and North Korea, while cultivars Tropical and cv. Doko were the oldest resistant Brazilian materials (1980s). PI 090763 from China (1930s), PI 196170 (South Korea), accessions from Japan (1950s), cv. Santa Rosa (1957), and the American cultivars Bragg and Davis (1960s) were examples of the oldest susceptible materials in this panel.

### Identification and mapping of the southern stem canker resistance locus

**Fig. 2:**
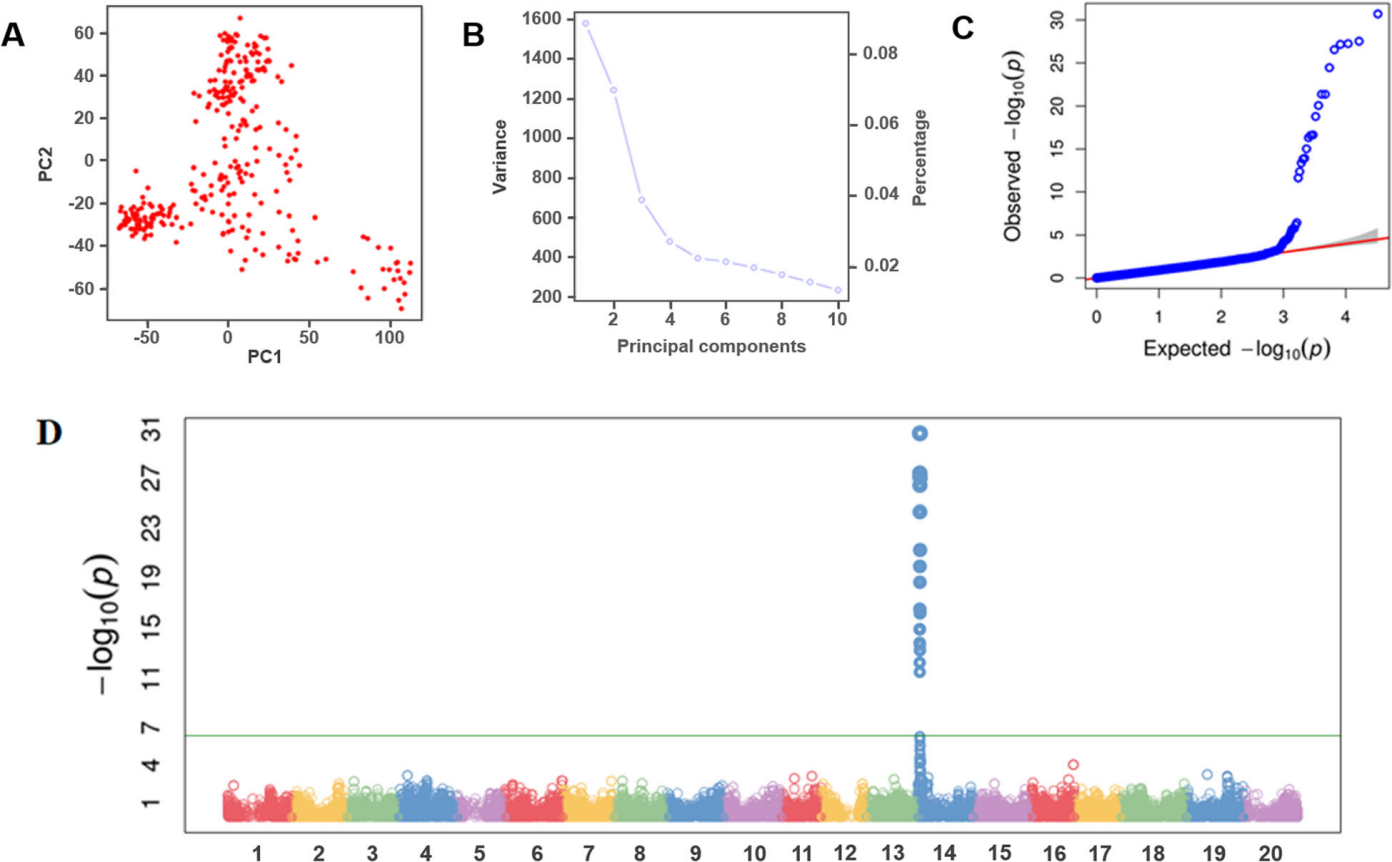
Manhattan plot, Quantile-quantile (QQ) plots and PCA of population structure for southern stem canker. **a** Principal component analysis of the GBS panel. **b** The genetic variation explained using 3 PCs. **c** QQ-plot from this GWAS. **d** Manhattan plot obtained from GWAS

The Fast-GBS pipeline produced approximately 50,000 high-quality SNPs from the GBS data. Using an MAF of ≥0.05 as a cut-off, we selected a total of 32,836 polymorphic SNP markers that we used in GWAS. The resulting SNPs were distributed over the whole genome. These SNPs proportionally covered all soybean chromosomes, with a mean SNP density of one SNP every 29.1 Kbp and a mean of 1642 SNP markers per chromosome. The greatest number of SNPs was detected on chromosome 18 (2845 SNPs), followed by chromosome 4 (2145 SNPs), and the lowest numbers were observed on chromosomes 12 (951 SNPs) and 11 (959 SNPs) (Additional file 1**)**. Regarding population structure, a principal component analysis (PCA) was performed, in which PC1 explained approximately 9% of the observed variance, PC2 approximately 7% and PC3 approximately 4%; together, the three PCs explained approximately 20% of the total genetic variance (Fig. 2a and b). The GWAS was performed with the compressed mixed linear model (cMLM), which accounted for population structure (PCA) and relatedness by the kinship matrix (K matrix). The quantile-quantile plot showed that the observed *p*-values strongly deviated from the expected *p*-values for a few SNPs, which indicated that the cMLM model was appropriate for the performed GWAS (Fig. 2c). We identified a single locus on chromosome 14 at which a total of 19 SNPs showed significant associations (FDR < 0.001) with SSC resistance (Fig. 2d). Among these significant SNPs, the FDR-adjusted *p*-value ranged between 6.35E-27 and 4.13E-09, with SNPs explaining approximately 40 to 70% of the total phenotypic variation (Table 2).

**Table 2:** The most significant SNPs associated with SSC resistance identified in this study

Marker ID	Chrom.	Pos (bp)	MAF	*p*.value	*r* ^*2*^	FDR Adjusted *p*-values
GBSRdm370	14	1,744,370	0.30	1.93E-31	0.70	6.35E-27
GBSRdm556	14	1,725,556	0.24	2.97E-28	0.64	4.88E-24
GBSRdm287	14	1,710,287	0.28	5.61E-28	0.63	5.66E-24
GBSRdm224	14	1,986,224	0.27	6.89E-28	0.63	5.66E-24
GBSRdm562	14	1,740,562	0.25	2.87E-27	0.62	1.89E-23
GBSRdm793	14	1,768,793	0.42	3.66E-25	0.59	2.00E-21
GBSRdm339	14	1,921,339	0.28	4.17E-22	0.54	1.71E-18
GBSRdm374	14	1,921,374	0.28	4.17E-22	0.54	1.71E-18
GBSRdm219	14	1,795,219	0.45	8.48E-21	0.52	3.09E-17
GBSRdm204	14	1,751,204	0.21	1.60E-19	0.50	5.27E-16
GBSRdm516	14	1,612,516	0.27	2.26E-17	0.47	6.75E-14
GBSRdm964	14	1,850,964	0.40	2.57E-17	0.47	7.02E-14
GBSRdm114	14	1,851,114	0.40	4.80E-17	0.46	1.21E-13
GBSRdm450	14	1,612,450	0.26	9.59E-16	0.45	2.25E-12
GBSRdm397	14	1,612,397	0.23	1.19E-14	0.43	2.60E-11
GBSRdm518	14	1,744,518	0.46	1.52E-14	0.43	3.13E-11
GBSRdm120	14	1,741,120	0.45	4.36E-14	0.42	8.43E-11
GBSRdm712	14	1,581,712	0.23	4.26E-13	0.41	7.77E-10
GBSRdm875	14	1,581,875	0.32	2.39E-12	0.40	4.13E-09

*Chrom.* Chromosome, *Pos (bp)* physical position of the allelic variant, *MAF* Minor allele frequency, *r*^*2*^ R squared value of the model with the SNP. All SNPs were physically positioned in the Wm82.a2 version of the Glycine max genome

The interval delimited by the significant SNPs extended just over 400 kbp, although the three most significant SNPs were located within a span of 34 kbp, thus identifying a very specific region. Within this region, the most significant SNP resided within *Glyma.14 g024300* (a DEA(D/H)-box RNA helicase family protein), the second most significant SNP resided within *Glyma.14 g024100* (a Rho GTPase-activating protein), and the third most significant SNP was located within *Glyma.14 g23900* (a methionine sulfoxide reductase).

Based on the results, the peak SNP by itself was sufficient to separate the resistant and susceptible accessions with a high level of concordance. At the peak SNP (1,744,370 – SNP1), the C allele was detected in 194 resistant accessions, while four resistant accessions were heterozygous, and the remaining seven resistant accessions showed the T allele. Similarly, an elevated concordance between the phenotype and genotype was observed among the susceptible materials. Among 90 susceptible accessions, 71 showed the T allele. Of the 19 apparent discrepancies, 16 accessions were heterozygous, and the remaining three carried the C allele. A comprehensive description of the SNP genotypes (at all 19 significant positions) and phenotypes for each accession is provided in Additional file 2.

Among the differential accessions, the C allele was detected at the peak SNP in all accessions that showed resistance to isolate CMES 480 as well as in the susceptible accession D85–10404, which is a line derived from cv. Tracy-M. On the other hand, cv. Dowling and the D85–10412 line showed both the susceptible phenotype and the T allele (Additional file 3).

We performed a haplotype analysis of the 295 accessions using SNPs associated with SSC resistance. First, from the initial 19 SNPs showing significant associations, we eliminated redundant SNPs (i.e., SNPs associated with SSC that provided the same information). Thereafter, we obtained four haplotypes containing the combination of four SNPs that were able to discriminate the main SSC resistance sources and grouped the accessions presented in the panel (Table 3). Haplotype 1 was present in the majority of resistant materials and was shared by cv. Hutcheson and the PI 398469 and was present in just one susceptible accession. Haplotype 2 was shared only by cv. Crockett and 35 resistant accessions. Haplotype 3, shared by cv. Tracy-M and line D85–10404, was also present in 22 resistant and two susceptible accessions. Finally, haplotype 4 was distributed in 70 susceptible accessions, in Dowling and line D85–10412 and in 5 other resistant accessions.

**Table 3:** Haplotypes obtained using SNPs from GWAS for the accessions

Haplotype ID		SNPs Positions In The Soybean Genome				SSC Phenotype	
	Differential Genotypes	1,744,370	1,768,793	1,744,518	R	S	Total
Hap1	Hutcheson/PI 398469	C	C	C	124	1	125
Hap2	Crockett	C	C	A	36	0	36
Hap3	Tracy-M/D85–10404	C	G	A	23	2	25
Hap4	Dowling/D85–10412	T	G	A	5	70	75

SNP positions: physical positions of allelic variants on chromosome 14 of the soybean genome (Wm82.a2); R: SSC-resistant accessions; S: SSC-susceptible accessions. In the haplotype analyses, only accessions showing homozygous alleles for all three SNPs were considered

### Whole-genome sequencing in the resistance locus interval reveals additional allelic variation

Analysis of the region associated with resistance against *Da* was performed by examining allelic variation 278 kb upstream and 200 kb downstream of the first peak SNP of the GWAS in the resequencing soybean dataset. This specific interval was based on SNPs with *r*^*2*^ values higher than 0.3, according to the LD analysis. (Additional file 4). We observed a total of 4440 SNPs and 1105 InDels in this interval (Table 4). Among the SNPs, 3375 were identified in noncoding regions, 421 in intronic regions, 247 in UTRs, and 397 in exons. Among the last group, 248 nonsynonymous SNPs were observed in 39 different genes. Moreover, there were 69 InDels in UTRs, 98 InDels in introns, and 37 InDels in exons. Twenty-three InDels were responsible for a frameshift modification in 9 different genes.

**Table 4:** Summary of the allelic variation observed in the putative Rdm locus region

Region		Modification	SNPs	InDels
Non-Coding		Intergenic region	143	14
		Upstream 5 k	2537	683
		Downstream 5 k	695	204
Coding	UTR	5′ UTR	88	23
		5′ UTR premature start gained	8	–
		3′ UTR	151	46
	Intron	Intron	399	96
		Splice region	19	2
		Splice acceptor site	2	–
		Splice donor site	1	–
	Exon	Disruptive + In-frame Deletion	–	3
		Disruptive + In-frame Insertion	–	2
		Frameshift	–	23
		In-frame Deletion	–	5
		In-frame Insertion	–	4
		Nonsynonymous modification	248	–
		Start lost	1	–
		Stop retained	1	–
		Stop gained	5	–
		Synonymous modification	142	–
Total			4440	1105

Intergenic region: the variant is in an intergenic region; Upstream 5 k: SNPs detected up to 5 kb upstream of the coding region; Downstream 5 k: SNPs detected up to 5 kb downstream of the coding region; 5′ UTR: hits in the 5’UTR; 5′ UTR premature start gained: a variant in the 5’UTR produces a three-base sequence that can be a START codon; 3′ UTR: variant hits in the 3’UTR; Intron: SNPs detected within an intron; Splice region: a sequence variant in which a change has occurred within the region of the splice site, either within 1–3 bases of the exon or 3–8 bases of the intron; Splice acceptor site: the variant hits a splice acceptor site; Disruptive + In-frame Deletion: one codon is changed, and one or more codons are deleted; Disruptive + In-frame Insertion: one codon is changed, and one or many codons are inserted; Frameshift: insertion or deletion causes a frameshift; In-frame Deletion: one or many codons are deleted; In-frame Insertion: one or many codons are inserted; Start lost: variant causes start codon to be mutated into a nonstart codon; Stop G: variant causes a STOP codon; Nonsynonymous modification: SNP variants cause a codon that produces a different amino acid; within the coding region; Synonymous modification: variant causes a codon that produces the same amino acid

**Fig. 3:**
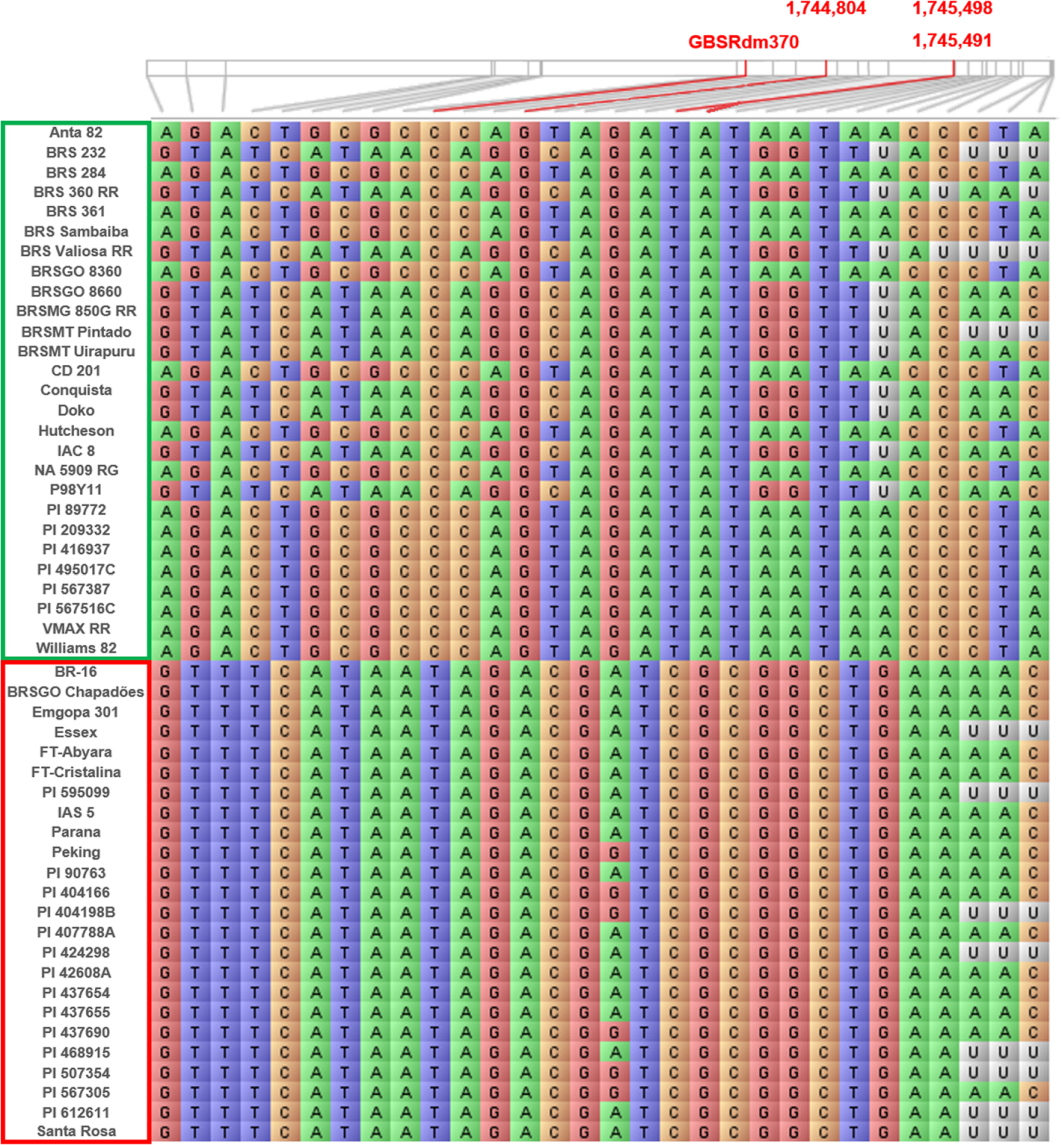
The allelic variation observed in 51 resequenced soybean cultivars for GBSRdm370 in this study. The soybean accessions in green squares represent the resistant lines, while the soybean accessions in red squares represent the susceptible lines

The most significant SNP was a nonsynonymous modification located at exon 6 of the *Glyma.14G024300* gene (encoding a DEAD/DEAH box RNA helicase). We also identified three other nonsynonymous SNPs associated with this gene (Fig. 3), which were in perfect LD with the first peak SNP and could not be detected by the GBS strategy due the lower coverage of the technique compared to whole-genome sequencing. Unsurprisingly, given the large size of the haplotype block comprising the peak SNP, we observed 216 SNPs and 46 InDels in perfect LD (*r*^*2*^ = 1) with the first peak SNP of the GWAS, at a distance up to 224 Kbp from the described allele (Additional file 4). Some of these allelic variations were distributed within genes in the interval that presented structural domains commonly found in resistance genes, revealing other potential candidate genes for SSC resistance. Fifteen nonsynonymous SNPs were observed in eight genes, including two leucine-rich-repeat receptor-like protein kinases (LRR-RPK) (*Glyma.14G026300*, and *Glyma.14G026500*), a serine-threonine protein kinase (PRSTK) (*Glyma.14G026700*), a PH domain LRR-containing protein phosphatase 1 (*Glyma.14G024400*), a methyltransferase (*Glyma.14G026600*), an acid phosphatase-related gene (*Glyma.14G024700*), and a gene involved in DNA repair (*Glyma.14G026900*) (Table 5). Finally, an insertion of two nucleotides responsible for a frameshift modification in the exon of an LRR-RPK gene (*Glyma.14G026500*) was observed only in susceptible cvs. Based on our analysis. To confirm the association of these allelic variations and the role of potential candidate genes in resistance to SSC, functional validation should be conducted in future studies.

**Table 5:** Fifteen nonsynonymous mutations with similar patterns of GBSRdm370 detected in the haplotype analysis

Pos	REF	ALT	Ncl	AA	Gene	Exon
1747,030	G	A	Gag/Aag	p.Glu8Lys/c.22G > A	Glyma.14G024400	1
1747,238	C	G	gCt/gGt	p.Ala77Gly/c.230C > G	Glyma.14G024400	1
1747,247	C	T	tCg/tTg	p.Ser80Leu/c.239C > T	Glyma.14G024400	1
1747,286	G	A	aGg/aAg	p.Arg93Lys/c.278G > A	Glyma.14G024400	1
1747,288	G	A	Gaa/Aaa	p.Glu94Lys/c.280G > A	Glyma.14G024400	1
1,783,495	A	G	Acc/Gcc	p.Thr417Ala/c.1249A > G	Glyma.14G024700	5
1,887,464	G	A	aGc/aAc	p.Ser4Asn/c.11G > A	Glyma.14G026300	1
1,908,059	G	A	Ggt/Agt	p.Gly285Ser/c.853G > A	Glyma.14G026500	3
1,908,548	T	G	tgT/tgG	p.Cys336Trp/c.1008 T > G	Glyma.14G026500	4
1,909,357	T	C	Atg/Gtg	p.Met274Val/c.820A > G	Glyma.14G026600	9
1,909,360	T	A	Atc/Ttc	p.Ile273Phe/c.817A > T	Glyma.14G026600	9
1,912,650	A	G	Tct/Cct	p.Ser44Pro/c.130 T > C	Glyma.14G026600	1
1,915,601	G	A	Gcc/Acc	p.Ala31Thr/c.91G > A	Glyma.14G026700	1
1,917,088	T	G	Tat/Gat	p.Tyr193Asp/c.577 T > G	Glyma.14G026700	5
1,942,282	C	T	cCt/cTt	p.Pro558Leu/c.1673C > T	Glyma.14G026900	3

*Pos* position of the identified SNP in base pairs (bp), *REF* Allele corresponding to the reference genome (Williams 82), *ALT* Alternative allele observed at this position, *Ncl* Nucleotide modification observed due this SNP, *AA* Amino acid modification observed due this SNP, *Exon* The exon of the gene in which the SNP was identified

### Allelic discrimination using the *Rdm* SNP KASP assay

**Fig. 4:**
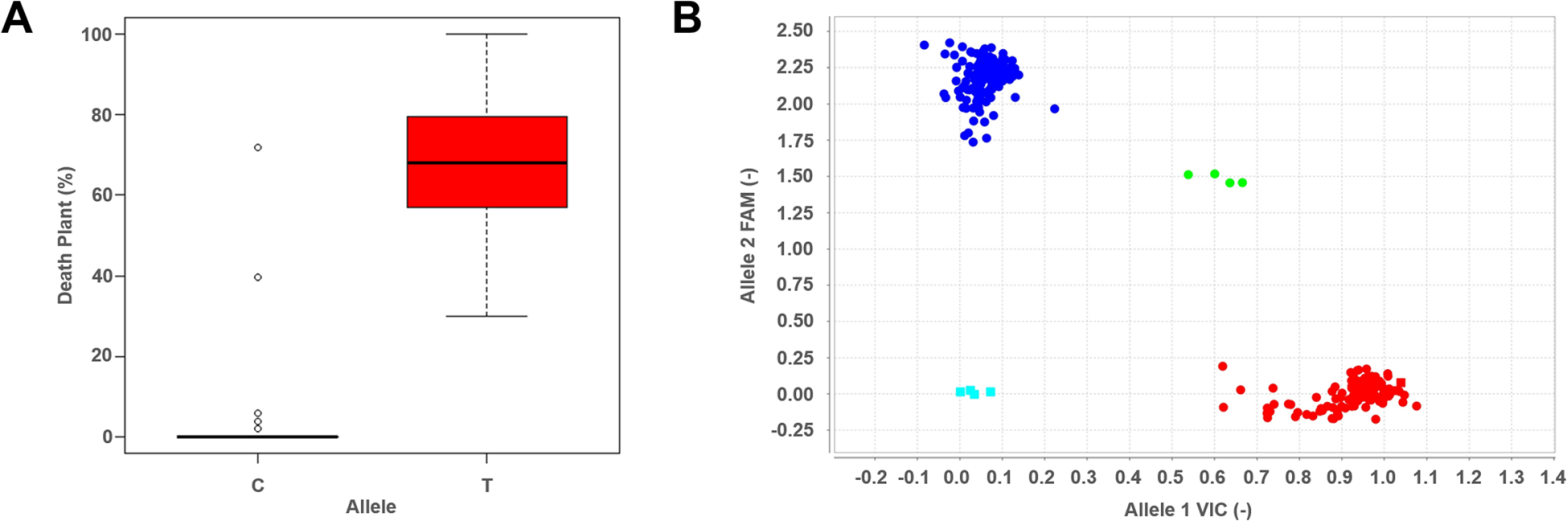
Box plot and allelic discrimination of the GBSRdm370 SNP. **a** Box plot of the GBSRdm379 markers associated with %DP. **b** Allelic discrimination observed GBSRdm370. The blue dots represent the resistance allele; the red dots represent the susceptible allele; and the green dots represent the heterozygous samples

The peak SNP (1,744,370) was selected to develop a KASP assay to confirm the alleles obtained by GBS and to apply this assay in future MAS. Thus, a subset of 146 accessions from the GWAS panel were analyzed with this assay, and as expected, all of the same alleles/genotypes obtained by GBS were obtained using the KASP assay (Additional file 5). Furthermore, the developed assay was able to correct the heterozygous genotypes obtained by GBS (Fig. 4). Among the accessions shown to be heterozygous at the peak SNP, 15 accessions were present in the subset analyzed with the assay, and all were found to be homozygous.

Therefore, the efficiency of the SNP marker and type I/II error rates were calculated and are shown in Table 6. The SNP1 marker was present in 98% of the accessions phenotyped as resistant, resulting in a low type I error rate (2.4%), which suggests a low probability of erroneously selecting a susceptible line based on the marker genotype. In addition, the marker also presented a low type II error rate or false negative rate of 1.19%.

**Table 6:** Analysis of the agreement between genotyping and phenotyping using the CMES 480 isolate

SNP	Pos (bp)	Accuracy (%)	Recall (%)	Type I Error Rate (%)	Type II Error Rate (%)	
GBSRdm?370	1,744,370	98.0	98.0	2.4	1.18	

*Pos* Position of the identified SNP in base pairs (bp); Accuracy (%):the percentage of resistant and susceptible genotypes correctly classified by the marker; Recall (%): a value given by the number of true positives divided by the number of true positives plus the number of false negatives; Type I error rate (%): false-positive rate; Type II error rate (%): false-negative rate

## Discussion

### Southern stem canker reactions in the GWAS panel

Resistance to southern stem canker is an important trait for the release of a new soybean cultivars, considering that this disease presents a high potential to cause losses of up to 100% in soybean fields [8]. Almost all soybean cultivars currently registered in Brazil and in other countries are resistant to southern stem canker. However, few genetic studies have documented the main sources of resistance present in soybean cultivars. Regarding the Brazilian cultivars, there are no genetic studies showing the main SSC resistance sources present in Brazilian germplasms.

Considering the importance of SSC in Brazil, Brumer et al. recently characterized a Brazilian collection of isolates of the pathogen comprising samples collected in different regions and years and demonstrated the occurrence of at least three different races in Brazil [28]. Only the sources Tracy-M (*Rdm1*/*Rdm2*) and cultivar Crockett (*Rdm3*) showed a resistance reaction for all isolates in that study; thus, these genes have become targets for plant breeding programs. Given that our lack of knowledge about the main sources in our GWAS panel, the isolate CMES 480 was selected for our phenotyping approach due to showing incompatible reactions when inoculated onto the main SSC resistance source (the cultivars Tracy-M, Crockett, Hutcheson and PI 398469).

In the present study, the applied method was toothpick inoculation, which has been used successfully in the evaluation of soybean materials since the first outbreaks of the disease in the late 1980s [8, 13, 26, 28]. In our panel, 205 accessions were classified as resistant by this inoculation method, including the differential genotypes such as cv. Tracy-M, cv. Crockett, cv. Hutcheson and PI 398469, confirming their resistance determined in other studies [8, 10–12, 29–33]. Therefore, good reproducibility of this approach for assessing the correct SSC phenotype in the accessions was demonstrated, which is a crucial step for obtaining confident results in GWAS.

### Genome wide association study for southern stem canker disease

Using an MAF of 5%, we filtered approximately 36 K SNPs from the initial SNP data, which were used in the GWAS. The SNPs were distributed on all the soybean chromosomes, and as expected, a larger number of SNPs were detected on the largest chromosomes, as seen on chromosome 18. On the other hand, a smaller number of SNPs were detected on the smallest chromosomes, such as chromosome 11. Very similar SNP distribution patterns were obtained in recent GWASs for resistance to *Sclerotinia sclerotiorum* [19] and *Meloidogyne incognita* [34].

The GWAS conducted in the present work revealed a highly significant association of resistance to SSC with a 478 kbp region on chromosome 14. Therefore, we may assume that the main SSC resistance present in our panel is related to this region, although previous genetic mapping studies have detected other loci involved in SSC resistance, and we have used an isolate that is even able to select different R genes. In the present study, we used CMES 480, which selects different R genes; thus, we cannot assume that the peak SNP on chromosome 14 is associated with the resistance locus in all the accessions. Indeed, some accessions showed resistance derived from other R genes located in other genomic regions.

A similar region on chromosome 14 was recently identified by a GWAS conducted with SNPs from the SoySNP50K array and using phenotype information from the USDA Germplasm Bank [35]. In that study, it was also identified two SNPs associated with resistance to SSC caused by *D. aspalathi and D. caulivora* on chromosome 14 in a region spanning approximately 400 kb. However, it was previously demonstrated that the *Rdm1*-*Rdm5* genes that confer resistance to *D. aspalathi* do not confer resistance to *D. caulivora* [13], leading to the assumption that the region might contain different R genes for both *D. aspalathi* and *D. caulivora*. In our study, all accessions were screened for SSC resistance in the same experiment with the pure isolate of *D. aspalathi* previously characterized both morphological and molecularly [28]. The SNP (ss715617869) previously identified as related to SSC resistance [35] is located at 1,731,256 bp on chromosome 14, while the three peak SNPs detected in our association analysis are located in the interval between 1,710,287-1,744,370. Therefore, our SNPs overlapped with the region identified by Chang et al. [35], suggesting that the region identified in both studies is related to SSC caused by *D. aspalhati*.

Interestingly, although the peak SNP was present in almost all the SSC sources, the identified haplotype was able to differentiate the main resistance sources, leading to inferences about the origin of the R gene conferring resistance in the accessions. Most of the resistant materials in the panel shared the haplotype of cvs. Hutcheson and PI 398469 (Additional file 2). Therefore, we may assume that the form of SSC resistance in this panel is the same as that in these sources. In contrast, using *D. aspalhati* isolates and F_2:3_ populations derived from cv. Hutcheson, Chiesa et al. [15] reported the genetic mapping of *Rdm4 and Rdm5* on chromosome 8, indicating different regions conferring resistance in this source. The use of different isolates in each study (i.e., isolates selected for different R genes) and the differences in panel composition are the main explanations for this difference because they have direct consequences for the regions identified in the mapping studies. Similarly, other sources such as cv. Crockett and cv. Tracy-M showed specific haplotypes, and a considerable portion of the resistant accessions were grouped in these haplotypes, leading to the assumption that these accessions probably harbor the same resistance source shared by these cultivars.

Other studies have shown the success of haplotype analysis for discriminating resistance sources in soybean. Pham et al. [36] performed fine mapping of resistance to *Cercospora sojina* K. Hara in two accessions and constructed a haplotype using 11 SoySNP50K SNPs in the known resistance source (cv. Davis) and 45 lines and cultivars and obtained a haplotype unique to these two resistant accessions. Furthermore, they analyzed haplotype allele variation at the *Rcs3* locus (a *C. sojina* resistance gene) in the same accession panel. It was observed that the Davis haplotype was shared with only four cultivars and not by the two resistant accessions, which suggested that all the cultivars with the Davis haplotype may harbor the same resistance sources and confirmed the resistance haplotype unique to the other two accessions. In another recent study, King et al. [37] mapped the *Rpp4-b* locus in PI 423971 and used five SoySNP50K SNPs to construct the *Rpp4-b* haplotype, which was unique to PI 423971 and just four lines, while all other *Rpp* source genotypes and 32 susceptible soybean ancestors did not exhibit this haplotype. Then, the authors suggested that these lines may possess the *Rpp4-b* locus. Altogether, these studies and our results demonstrate the applicability of haplotype analysis for obtaining initial information about resistance sources and the possibility of discriminating these sources.

Considering that some Brazilian *D. aspalathi* isolates are able to cause disease in cv. Hutcheson and PI 398469 [28] but not in the cv. Crocket and cv. Tracy-M, it is possible that the SNPs associated with SSC on chromosome 14 might be linked with one or more *Rdm* genes in the region; however, to confirm this hypothesis, a further fine mapping study needs to be conducted in a biparental population obtained from independent crosses with these resistance sources. Therefore, we chose to designate this locus as a common locus for resistance to southern steam canker present in many different soybean accessions evaluated in this study. Furthermore, based on our results, the KASP assay using the most significant SNP associated with SSC in soybean can be considered useful to breeding programs for the marker-assisted selection of SSC resistance.

### New allelic variations based on resequencing analysis of soybean genomes

To confirm our results, we examined nucleotide variation on the basis of whole-genome resequencing data from a collection of 51 accessions that were characterized for their reaction to SSC isolates. The SNP haplotypes in the vicinity of the SNPs shown to be significantly associated with *Da* resistance in the GWAS were again clearly associated with the disease reaction.

The most significant SNP associated with SSC resistance based on GWAS was identified in *Glyma.14G024300*, a DEAD/DEAH box RNA helicase described as being involved in important biological processes such as transcription, translation initiation, mRNA splicing and export, and ribosome biogenesis [38–41]. A large number of studies have associated DEAD-box RNA helicases with different stresses in soybean, such as salt stress [38, 42], cold tolerance [38, 43], and resistance to a fungal pathogens [44].

Moreover, we identified allelic variations in perfect LD with SNP1 in LRR-RPK genes (*Glyma.14G026300*, and *Glyma.14G026500*). In *Arabidopsis thaliana*, several studies have associated LRR-RPK genes with defense mechanisms. An LRR-RPK gene has been described as a positive regulator of the ABA response during the stress response and plant development [45]. Another study in *Arabidopsis* showed that the ERECTA gene, previously described as being associated with development pathways, was also related to resistance against bacterial blight [46]. In soybean, some studies have associated LRR-RPK genes with stress. It has been observed in *Glycine soja* that overexpression of the GsLRPK gene contributes to an increase in tolerance to cold [47]. Finally, an RNA-seq study of the *Rbs3* locus aided in the identification of some candidate genes associated with resistance against brown stem root, which included some LRR-RPK genes [48]. In addition to LRR-RPK genes, allelic variations have been also observed in PRSTK (*Glyma.14G026700)*. A plant-receptor-like serine/threonine kinase was one of the first genes cloned and associated with defense mechanisms and plays a key role in the signal transduction pathway in plants [49, 50]. The presence of PRSTK has been reported to be involved in the defense response due to the plant-pathogen interactions in some organisms, such as rice [51], *Arabidopsis thaliana* [52], and soybean [53, 54]. The existence of nonsynonymous SNPs or InDels in the coding regions of these genes associated with plant stress could clarify the plant defense mechanisms related to SSC resistance. Thus, the DEAD-box RNA helicases (*Glyma.14G024300*), LRR-RPK (*Glyma.14G026300*, and *Glyma.14G026500*), and PRSTK (*Glyma.14G026700*) genes might be interesting targets for future functional studies to determine the effects of these genes in soybean during *Da* infection.

## Conclusion

In this study, we identified and confirmed the location of an important locus related to SSC resistance in soybean. At least three important sources of resistance to SSC (PI 398469, cv. Hutcheson and cv. Crocket) presented the locus mapped on chromosome 14. The identified peak SNP was able to correctly distinguish the resistant accessions in the panel with high precision. The developed marker assay associated with the *Rdm* locus will be a useful tool in breeding programs for marker-assisted selection to identify accessions carrying the allele conferring resistance against infection by *D. aspalathi* and to follow its introgression. Our results demonstrated the relevance of the *Rdm* locus on chromosome 14 for the resistance to SSC in Brazilian cvs. For the first time. Additionally, we characterized a significant number of plant accessions and cvs. Sharing different resistance haplotypes, which can be exploited by breeders.

## Materials and methods

### Plant materials

The source material for the analysis comprised a set of 295 soybean accessions (Additional file 6) representing different maturity groups and various regions of origin, such as China, Japan, North and South Korea, Russia, the United States, India and Brazil. The panel included accessions carrying previously described resistance genes (in parentheses): cv. Tracy-M (*Rdm1/Rdm2*), D84–10404 (*Rdm1*), D84–10412 (*Rdm2*), cv. Crockett (*Rdm3*), cv. Dowling (*Rdm4*), cv. Hutcheson (*Rdm4/Rdm5*) and PI 398469 (*Rdm?)*, while the cultivar BR23 served as a susceptible control. The seeds were obtained from the Embrapa Soybean Germplasm Bank.

### Phenotypic evaluation for stem canker

The soybean accessions in the GWAS panel and the accessions subjected to WGS were infected with the CMES 480 isolate of *D. aspalathi* (collected in Rio Verde (GO) in 2001) and evaluated in a greenhouse at Embrapa Soybean in Londrina (PR, Brazil) in 2015. Phenotyping was conducted using the toothpick method with colonized mycelium as described by Keeling [26] and modified by Yorinori [27]. The experimental design was completely randomized with two replicates including 10 plants in each pot. In both phenotyping trials, all inoculations were carried out on 10- to 15-day-old seedlings that were kept under high humidity (45-s nebulization every hour throughout the day), with average temperatures of 26 ± 4 °C (day) and 17 ± 3 °C (night). As a negative control, cv. BR 23 was inoculated with sterile toothpicks without mycelium. The evaluation of each genotype was performed 60 days after inoculation by counting the number of dead plants (DPs). The percentage of DPs (% DP) was calculated according to the method described by Yorinori [27]: %DP = {[DP + (IP/2)]/TP}*100, where IP is the total number of infected plants, and TP is the number of inoculated plants.

The accessions were classified based on plant-fungus interaction reactions described by Yorinori [27] and modified by Pioli et al. [13] into two categories: i) incompatible or avirulent (0–14.9% DP), which means that accession was considered resistant to the isolate; and ii) compatible (> 15% DPs), meaning that plants were classified as susceptible to SSC.

### DNA extraction and GBS library preparation

DNA was extracted using 100 mg (wet weight) of young leaf from a unique plant for each soybean accession with the DNeasy Plant Mini Kit (Qiagen Inc., Valencia, CA, USA) according the manufacturer’s instructions and subsequently quantified using a Nanodrop 8000 spectrophotometer (Thermo Fischer Scientific Inc., Waltham, MA, USA). Then, the samples were diluted to 10 ng/μl. The GBS libraries were constructed using the *Ape*KI restriction enzyme according to the protocol described by Elshire et al. [55], as modified by Sonah et al. [56]. Briefly, the DNA samples were digested with *Ape*KI enzyme, the fragments were selected by size, PCR reactions to include barcodes to identify each sample were performed and the pooling of the samples were carryout out. A subset of the resulting single-end sequencing of multiplex GBS libraries was sequenced on the Illumina HiSeq2000 platform (McGill University-Genome Quebec Innovation Center, Montreal, QC, Canada) and another set via Ion Torrent sequencers (IBIS - Institute of Integrative Biology and Systems, Université Laval, Quebec City, QC, Canada).

### SNP identification and GWAS

Illumina and Ion Torrent read processing, demultiplexing of samples, mapping in reference genome, SNP/indel calling and genotyping were performed by the Fast-GBS pipeline using the Williams 82 assembly 2 (Wm82.a2) [56]. Any heterozygous calls were replaced with missing data, and only SNPs with less than 80% missing data were retained. Indels were not used in the downstream analyses. Imputation of missing data was performed using Beagle [57]. Marker-trait associations were calculated with the GAPIT R package [58] using a compressed mixed linear model (cMLM). To control for population structure and relatedness between individuals, we used the first three principal components (PCs) obtained from principal component analysis (PCA) and the VanRaden kinship matrix in the GWAS model. We declared SNPs to be significant at an FDR-adjusted *p*-value of less than 0.001.

### Haplotype analysis and linkage disequilibrium detection

First, we performed haplotype analysis on the GWAS panel using the set of 19 SNPs that were most highly associated with SSC resistance in the GWAS. Then, we removed the redundant SNPs, and the haplotypes of the differential lines were constructed; haplotypes accounting for most of the resistant accessions were obtained. We carried out an analysis of linkage disequilibrium (LD) decay using the GBS-derived SNP dataset from the GWAS panel with the PopLDdecay 3.30 software package, and LD was measured using the squared allele frequency correlations (*r*^*2*^).

Furthermore, we investigated the allelic variation present in a subset of 51 accessions comprising 27 Brazilian soybean cvs [59]. and 23 other accessions from the center of origin [24] as well as PI 595099 and Williams 82 (reference genome) for the putative resistance locus mapped in this study using WGS data (Additional file 7). We performed LD analysis to identify SNPs associated with the peak SNP identified by GWAS. We used TASSEL software to generate *r*^*2*^ values and to determine which SNPs were in LD with the peak SNP. Finally, we used SnpEff [60] to detect SNPs associated with candidate genes in the soybean genome. The focus of this analysis was the allelic variation within genes located within the region identified based on GWAS. Graphical genotype visualization was performed using Flapjack [61].

### SNP assay design and genotyping

For the development of markers to be used for high-throughput genotyping, the peak SNP identified in the GWAS was selected, and a Kompetitive Allele Specific PCR (KASP) assay was designed. For SNP marker validation, a subset of the GWAS panel comprising 146 resistant and susceptible accessions was selected, including the seven differential lines [Tracy-M (*Rdm1*/*Rdm2*), D85–10404 (*Rdm1*), D85–10412 (*Rdm2*), Crockett (*Rdm3*), Dowling (*Rdm4*), Hutcheson (*Rdm4*/*Rdm5*) and PI 398469 (*Rdm?)*], (Additional file 5). DNA extraction was carried out using the DNeasy Plant Mini Kit. Briefly, for the KASP assay, the final volume of the reaction was 5.07 μL, containing 2.5 μL of diluted DNA (10 ng/ul), 1x KASP master mix, and 0.0014x KASP assay mix. SNP genotyping was performed using an ABI7900 instrument following a touchdown thermal cycling protocol described by the manufacturer. Genotypes were acquired and clustered using TaqMan Genotyper Software v2.1 (Life Technologies, Applied Biosystems Inc.; Foster City, CA, USA).

## Supplementary information

**Additional file 1.** Single nucleotide polymorphism distribution on soybean chromosomes.

**Additional file 2.** Haplotype analysis of the 295 accessions used in GWAS.

**Additional file 3.** Haplotype analysis of the eight differential Rdm sources of resistance.

**Additional file 4 **Linkage disequilibrium analysis for the *Rdm* locus region.

**Additional file 5.** List of soybean genotypes used for KASP assay validation.

**Additional file 6.** Basic description of the 295 soybean genotypes used in this study.

**Additional file 7.** The resequenced 51 soybean accessions used for haplotype analysis.

## Data Availability

All sequence reads described in the manuscript are available at DDBJ/EMBL/GenBank under BioProjects accession PRJNA294227, and PRJNA289660.

## Abbreviations

bp: base pair
cMLM: compressed Mixed Linear Model
CNVs: Copy Number Variations
cv.: cultivar
*Da*: *Diaporthe aspalathi*
*Dc*: *Diaporthe caulivora*
DP: Dead Plants
*Dpc*: *Diaporthe phaseolorum* var. *caulivora*
*Dpm*: *Diaporthe phaseolorum* var*. meridionalis*
GWAS: Genome-Wide Association Analysis
IP: Infected Plants
kbp: kilobase pair
LD: Linkage Disequilibrium
LRR-RPK: Leucine-rich-repeat receptor-like protein kinase
mAF: minor Allele Frequency
MAS: Marker-Assisted Selection
Mbp: Megabase pair
PRSTK: Plant-receptor-like serine/threonine kinase
QTLs: Quantitative Trait Loci
SNPs: Single Nucleotide Polymorphisms
SSC: Southern Stem Canker
TP: Total Plants

## References

[1] Lee G-A, Crawford GW, Liu L, Sasaki Y, Chen X (2011). Archaeological soybean (*Glycine max*) in East Asia: does size matter?. PLoS One.

[2] Companhia Nacional de Abastecimento. Séries Históricas de Área Plantada, Produtividade e Produção, Relativas às Safras 1976/77 a 2017/18 de Grãos, 2001 a 2017 de Café, 2005/06 a 2017/18 de Cana-de-Açúcar. 2018. http://www.conab.gov.br/conteudos.php?a=1252&. Accessed 2 Feb 2017.

[3] Benko-iseppon AM, Nepomuceno AL, Abdelnoor RV (2012). GENOSOJA – The Brazilian Soybean Genome Consortium : High throughput omics and beyond. Genet Mol Biol.

[4] Janse Van Rensburg JC, Lamprecht SC, Groenewald JZ, Castlebury LA, Crous PW (2006). Characterisation of *Phomopsis* spp. associated with die-back of rooibos (*Aspalathus linearis*) in South Africa. Stud Mycol.

[5] Santos JM, Vrandečić K, Ćosić J, Duvnjak T, Phillips AJL (2011). Resolving the diaporthe species occurring on soybean in Croatia. Persoonia Mol Phylogeny Evol Fungi.

[6] Udayanga D, Liu X, Crous PW, McKenzie EHC, Chukeatirote E, Hyde KD (2012). A multi-locus phylogenetic evaluation of *Diaporthe* (*Phomopsis*). Fungal Divers.

[7] Yorinori JT, Almeida AMR, Homechin M, Miranda LC, Kiihl RAS, Pola JN (1989). Epifitia do cancro da haste da soja nos municípios de Castro, Palmeira, Ponta Grossa e Tibagi no Paraná e Rondonópolis, no Mato Grosso, na safra de 1988/89. 5. Seminário Nacional de Pesquisa de Soja, Campo Grande MS.

[8] YORINORI JT. Cancro da haste da soja: epidemiologia e controle. Londrina: Embrapa Soja; 1996. p. 75.

[9] Wrather JA, Anderson TR, Arsyad DM, Gai J, Ploper LD, Porta-Puglia A (1997). Special report soybean disease loss estimates for the top 10 soybean producing countries in 1994. Plant Dis.

[10] Kilen TC, Hartwig EE (1987). Identification of single genes controlling resistance to stem canker in soybean. Crop Sci.

[11] Bowers GR, Ngeleka K, Smith O (1993). Inheritance of stem canker resistance in soybean cultivars Crockett and Dowling. Crop Sci.

[12] Tyler JM (1995). Additional sources of stem canker resistance in soybean plant introductions. Crop Sci.

[13] Pioli RN, Morandi EN, Martínez MC, Lucca F, Tozzini A, Bisaro V (2003). Morphologic, molecular, and pathogenic characterization of *Diaporthe phaseolorum* variability in the Core soybean-producing area of Argentina. Phytopathology.

[14] Chiesa MA, Pioli RN, Cambursano MV, Morandi EN (2013). Differential expression of distinct soybean resistance genes interacting with Argentinean isolates of *Diaporthe phaseolorum* var. *meridionalis*. Eur J Plant Pathol.

[15] Chiesa MA, Cambursano MV, Pioli RN, Morandi EN. Molecular mapping of the genomic region conferring resistance to soybean stem canker in Hutcheson soybean. Mol Breed. 2017;37:1-12.

[16] Schmutz J, Cannon SB, Schlueter J, Ma J, Mitros T, Nelson W (2010). Genome sequence of the palaeopolyploid soybean. Nature.

[17] Hwang E-Y, Song Q, Jia G, Specht JE, Hyten DL, Costa J (2014). A genome-wide association study of seed protein and oil content in soybean. BMC Genomics.

[18] Iquira E, Humira S, François B. Association mapping of QTLs for sclerotinia stem rot resistance in a collection of soybean plant introductions using a genotyping by sequencing (GBS) approach. BMC Plant Biol. 2015;5:1–12. 10.1186/s12870-014-0408-y PMC4304118 25595526

[19] Wei W, Mesquita ACO, Figueró AA, Wu X, Manjunatha S, Wickland DP, et al. Genome-wide association mapping of resistance to a Brazilian isolate of Sclerotinia sclerotiorum in soybean genotypes mostly from Brazil. BMC Genomics. 2017;18:1–16. 10.1186/s12864-017-4160-1 PMC5674791 29115920

[20] Mamidi S, Lee RK, Goos RJ, McClean PE (2014). Genome-wide association studies identifies seven major regions responsible for Iron deficiency Chlorosis in soybean (*Glycine max*). PLoS One.

[21] Mamidi S, Chikara S, Goos RJ, Hyten DL, Annam D, Moghaddam SM (2011). Genome-wide association analysis identifies candidate genes associated with Iron deficiency Chlorosis in soybean. Plant Genome J.

[22] Contreras-Soto RI, Mora F, de Oliveira MAR, Higashi W, Scapim CA, Schuster I (2017). A genome-wide association study for agronomic traits in soybean using SNP markers and SNP-based haplotype analysis. PLoS One.

[23] Zhou Z, Jiang Y, Wang Z, Gou Z, Lyu J, Li W (2015). Resequencing 302 wild and cultivated accessions identifies genes related to domestication and improvement in soybean. Nat Biotechnol.

[24] Valliyodan B, Dan Q, Patil G, Zeng P, Huang J, Dai L (2016). Landscape of genomic diversity and trait discovery in soybean. Sci Rep.

[25] Ramakrishna G, Kaur P, Nigam D, Chaduvula PK, Yadav S, Talukdar A (2018). Genome-wide identification and characterization of InDels and SNPs in *Glycine max* and *Glycine soja* for contrasting seed permeability traits. BMC Plant Biol.

[26] Keeling BL (1982). A Seedling Test for Resistance to Soybean Stem Canker Caused by *Diaporthe phaseolorum* var. *caulivora*. Phytopathology.

[27] Yorinori JT (1991). Metodologia para avaliação da resistência ao cancro da haste da soja. Fitopatol Bras.

[28] Brumer BB, Lopes-Caitar VS, Chicowski AS, Beloti JD, Castanho FM (2018). Gregório da Silva DC, et al. morphological and molecular characterization of *Diaporthe* (anamorph *Phomopsis*) complex and pathogenicity of *Diaporthe aspalathi* isolates causing stem canker in soybean. Eur J Plant Pathol.

[29] Chiesa MA, Pioli RN, Morandi EN (2009). Specific resistance to soybean stem canker conferred by the *Rdm4* locus. Plant Pathol.

[30] Siviero A, Menten JOM (1995). Uso de método do palito para inoculação de *Diaporthe* phaseolorum f. sp. *meridionalis* em soja. Summa Phytopathol.

[31] Gonçalves ECP, Centurion MAP d C, Di Mauro AO (2006). Avaliação de características agronômicas e de reação ao cancro-da-haste a ao oídio em linhagens de soja. Rev Ceres.

[32] Hillen T, Juliatti FC, Polizel AC, Hamawaki OT, de Brito CH (2006). Reação de genótipos de soja quanto à resistência ao cancro da haste. Biosci J.

[33] de Olliveira AL, Lam-Sanchez A (2005). Herança da resistência ao cancro da haste (*Diaporthe phaseolorum* ( Cke .& Ell ). Sacc . F . sp . *meridionalis* Morgan Jones ) em soja (*Glycine max* (L.) Merrill). Rev Nucl.

[34] Passianotto ALDL, Sonah H, Dias WP, Abdelnoor RV (2017). Genome-wide association study for resistance to the southern root-knot nematode (*Meloidogyne incognita*) in soybean. Mol Breed.

[35] Chang H, Lipka AE, Domier LL, Hartman GL (2016). Characterization of disease resistance loci in the USDA soybean Germplasm collection using genome-wide association studies. Genet Res.

[36] Pham AT, Harris DK, Buck J, Hoskins A, Serrano J, Abdel-Haleem H, et al. Fine mapping and characterization of candidate genes that control resistance to *Cercospora sojina K. Hara* in two soybean germplasm accessions. PLoS One. 2015;10. 10.1371/journal.pone.0126753 PMC4437980 25993056

[37] King ZR, Childs SP, Harris DK, Pedley KF, Buck JW, Boerma HR, et al. A new soybean rust resistance allele from PI 423972 at the *Rpp4* locus. Mol Breed. 2017;37.

[38] Chung E, Cho C, Yun B, Choi H, So H, Lee S (2009). Molecular cloning and characterization of the soybean DEAD-box RNA helicase gene induced by low temperature and high salinity stress. Gene.

[39] Rocak S, Linder P (2004). DEAD-box proteins: the driving forces behind RNA metabolism. Nat Rev Mol Cell Biol.

[40] Cordin O, Banroques J, Tanner NK, Linder P (2006). The DEAD-box protein family of RNA helicases. Gene.

[41] Linder P (2006). Dead-box proteins: a family affair--active and passive players in RNP-remodeling. Nucleic Acids Res.

[42] Sanan-mishra N, Pham XH, Sopory SK, Tuteja N (2005). Pea DNA helicase 45 overexpression in tobacco confers high salinity tolerance without affecting yield. Proc Natl Acad Sci.

[43] Gong Z, Dong C, Lee H, Zhu J, Xiong L, Gong D (2005). A DEAD Box RNA Helicase Is Essential for mRNA Export and Important for Development and Stress Responses in Arabidopsis. Plan.

[44] Li D, Liu H, Zhang H, Wang X, Song F (2008). OsBIRH1, a DEAD-box RNA helicase with functions in modulating defence responses against pathogen infection and oxidative stress. J Exp Bot.

[45] Osakabe Y, Maruyama K, Seki M, Satou M, Shinozaki K, Yamaguchi-shinozaki K (2005). Leucine-Rich Repeat Receptor-Like Kinase1 Is a Key Membrane-Bound Regulator of Abscisic Acid Early Signaling in Arabidopsis. Plant Cell.

[46] Godiard L, Sauviac L, Torii KU, Grenon O, Mangin B, Grimsley NH (2003). ERECTA, an LRR receptor-like kinase protein controlling development pleiotropically affects resistance to bacterial wilt. Plant J.

[47] Yang L, Wu K, Gao P, Liu X, Li G, Wu Z (2014). GsLRPK, a novel cold-activated leucine-rich repeat receptor-like protein kinase from *Glycine soja*, is a positive regulator to cold stress tolerance. Plant Sci.

[48] McCabe CE, Cianzio SR, O’Rourke JA, Graham MA (2018). Leveraging RNA-Seq to characterize resistance to Brown stem rot and the Rbs3 locus in soybean. Mol Plant-Microbe Interact.

[49] Martin GB, Brommonschenkel SH, Chunwongse J, Frary A, Ganal MW, Spivey R (1993). Map-based cloning of a protein kinase gene conferring disease resistance in tomato. Science (New York, NY).

[50] Zhou J, Loh YT, Bressan RA, Martin GB (1995). The tomato gene Pti1 encodes a serine/threonine kinase that is phosphorylated by Pto and is involved in the hypersensitive response. Cell.

[51] Song W-Y, Wang G-L, Chen L-L, Kim H-S, Pi L-Y, Holsten T (1995). A Receptor Kinase-Like Protein Encoded by the Rice Disease Resistance Gene, Xa21. Science.

[52] Gómez-Gómez L, Boller T (2000). FLS2: an LRR receptor-like kinase involved in the perception of the bacterial elicitor Flagellin in *Arabidopsis*. Mol Cell.

[53] Li Y, Sun S, Zhong C, Wang X, Wu X, Zhu Z (2017). Genetic mapping and development of co-segregating markers of RpsQ, which provides resistance to *Phytophthora sojae* in soybean. Theor Appl Genet.

[54] Ithal N, Recknor J, Nettleton D, Maier T, Baum TJ, Mitchum MG (2007). Developmental transcript profiling of cyst nematode feeding cells in soybean roots. Mol Plant-Microbe Interact.

[55] Elshire RJ, Glaubitz JC, Sun Q, Poland JA, Kawamoto K, Buckler ES (2011). A robust, simple genotyping-by-sequencing (GBS) approach for high diversity species. PLoS One.

[56] Sonah H, Bastien M, Iquira E, Tardivel A, Légaré G, Boyle B (2013). An improved genotyping by sequencing (GBS) approach offering increased versatility and efficiency of SNP discovery and genotyping. PLoS One.

[57] Scheet P, Stephens M, Tier B, van der Werf JH, Cleveland MA, Scheet P (2006). A fast and flexible statistical model for large-scale population genotype data: applications to inferring missing genotypes and Haplotypic phase. Am J Hum Genet.

[58] Lipka AE, Tian F, Wang Q, Peiffer J, Li M, Bradbury PJ (2012). GAPIT: genome association and prediction integrated tool. Bioinformatics.

[59] Maldonado dos Santos JV, Valliyodan B, Joshi T, Khan SM, Liu Y, Wang J (2016). Evaluation of genetic variation among Brazilian soybean cultivars through genome resequencing. BMC Genomics.

[60] Cingolani P, Platts A, Wang LL, Coon M, Nguyen T, Wang L (2012). A program for annotating and predicting the effects of single nucleotide polymorphisms, SnpEff: SNPs in the genome of *Drosophila melanogaster* strain w1118 ; iso-2; iso-3. Landes Biosci.

[61] Milne I, Shaw P, Stephen G, Bayer M, Cardle L, Thomas WTB (2010). Flapjack-graphical genotype visualization. Bioinformatics.

